# Profiling *Listeria monocytogenes* in Hummus, Fresh Produce, and Food Processing Environments in the Western Cape, South Africa

**DOI:** 10.1002/mbo3.70060

**Published:** 2025-09-08

**Authors:** Samantha Anne du Toit, Pieter A. Gouws, Diane Rip

**Affiliations:** ^1^ Department of Food Science Stellenbosch University Stellenbosch Western Cape South Africa

**Keywords:** antibiotic resistance, food safety, listeriosis, virulence

## Abstract

*Listeria monocytogenes* is pervasive in agricultural environments and difficult to eradicate from food‐processing facilities. Consequently, various foods become contaminated, posing health risks to immunocompromised individuals. This surveillance study aimed to enhance the understanding of the genetic diversity, virulence, plasmid content, sanitizer tolerance, and antibiotic resistance of *L. monocytogenes* from ready‐to‐eat (RTE) hummus, fresh produce and food‐processing environments in the Western Cape, South Africa (2018–2021). Sixty *L. monocytogenes* isolates were classified as lineage I or lineage II using polymerase chain reaction‐restriction fragment length polymorphism (PCR‐RFLP). Lineage I was notably prevalent overall (57%; *n* = 34) and significantly associated with fresh produce (88%; *n* = 7) (*p* = 0.04). Whole‐genome sequencing (WGS) and bioinformatic analysis characterized a subset of 20 *L. monocytogenes* isolates into seven sequence types (STs) (ST1, ST2, ST3, ST5, ST101, ST121, ST204), and three serotypes (1/2a, 1/2b, 4b). ST204 (38%; *n* = 3) was most prevalent in the food‐processing environment, ST5 (50%; *n* = 3) in fresh produce, and ST5 (33%; *n* = 2), ST101 (33%; *n* = 2) and ST121 (33%; *n* = 2), in RTE hummus. However, no single serotype or ST was significantly overrepresented in any category (*p* > 0.05). All isolates carried both *Listeria* pathogenicity island (LIPI)‐1 and LIPI‐2 gene clusters. Two isolates (ST1 and ST3) contained all genes comprising LIPI‐3. Two ST5 isolates from RTE hummus contained the *tetM* gene. Isolates from different origins contained the *emr*C, *bcr*ABC or *qac*H genes conferring tolerance to benzalkonium chloride, a quaternary ammonium compound class of sanitizer. RTE hummus, fresh produce and the food‐processing environment are susceptible to contamination by diverse and virulent *L. monocytogenes* strains.

## Graphical abstract

Minimally processed, ready‐to‐eat foods like hummus and fresh produce are associated with listeriosis, posing a significant risk to certain individuals. *Listeria monocytogenes* from these foods and production facilities contained key virulence genes, including those linked to hypervirulence and sanitizer tolerance. These findings highlight the importance of targeted food safety strategies and increased consumer awareness.

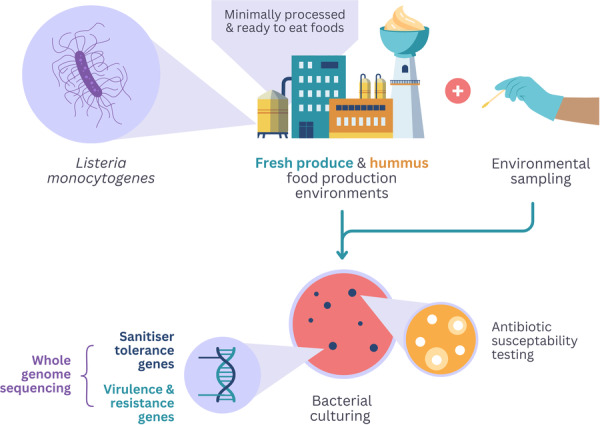

## 1 Introduction

*L. monocytogenes* causes listeriosis, a serious but rare disease acquired mainly by the consumption of contaminated foods (Nastasijevic et al. 2017; Skowron et al. 2018). The pathogen causes flu‐like symptoms in healthy individuals and life‐threatening, invasive infections in immunocompromised individuals, the elderly, pregnant women, and newborn babies (Chen et al. 2017). Although the incidence of listeriosis is usually low compared to other foodborne diseases, most cases require hospitalization (90%), and the disease outcome is often more serious (fatality rate of 20% to 30%) (Bouymajane et al. 2021; Chen et al. 2017; Hyden et al. 2016).

Listeriosis outbreaks associated with frozen corn (Maćkiw et al. 2021), packaged leafy salads (CDC 2015b; Self et al. 2019), bean sprouts (Garner and Kathariou 2016), stone fruit (CDC 2015a), caramel apples (Stephan et al. 2015), RTE salads (Gaul et al. 2013), cantaloupe (CDC 2015a) and celery (Olaimat et al. 2018a) have been reported since 2010, which encouraged surveillance of *L. monocytogenes* in fresh produce in South Africa. RTE hummus has also been responsible for listeriosis outbreaks (du Toit and Rip 2024), such as that experienced in California in 2013, with 28 cases, 25 hospitalizations and three deaths (Olaimat et al. 2018a; Salazar et al. 2020). However, *L. monocytogenes* from this food product is under‐investigated worldwide (du Toit and Rip 2024). Water activity and pH values of hummus and its main component, chickpeas, are conducive to the growth of *L. monocytogenes* (Salazar et al. 2020). This dip typically contains a high concentration of nutrients, which also promotes growth (Olaimat et al. 2018a).

The cultivation of fresh produce predisposes it to *L. monocytogenes* contamination, and contamination of food‐processing environments often leads to the contamination of RTE foods post‐processing (Garner and Kathariou 2016; Townsend et al. 2021). Fresh produce and RTE hummus are typically stored at refrigeration temperatures suited to the survival of *L. monocytogenes* and are consumed with minimal processing or no heat‐at‐home step (Bouymajane et al. 2021).

In 2017 and 2018, South Africa witnessed the largest listeriosis outbreak globally with 1060 cases and 216 deaths (Desai et al. 2019; NICD 2017; Smith et al. 2019a). A national surveillance system using WGS has since been implemented for all clinically confirmed listeriosis cases (Matle et al. 2020b). However, surveillance of *L. monocytogenes* from the food and agricultural industries in South Africa is insufficient. Very few South African studies have investigated the distribution and dominance of serotypes and sequence types (STs) in foods and food‐processing environments. Collecting surveillance data for foods less studied (or those not commonly considered high‐risk), throughout stages of food production supports a precautionary approach to food safety and helps mitigate the risk of listeriosis outbreaks (Smith et al. 2019b; Varma et al. 2007).

This study aimed to enhance the understanding of the genetic diversity, virulence, and resistance mechanisms of *L. monocytogenes* isolates from RTE hummus, fresh produce, and the food‐processing environment in the Western Cape, South Africa (2018–2021). The main objectives were to lineage type 60 *L. monocytogenes* isolates using PCR‐RFLP, to characterize 20 *L. monocytogenes* isolates into STs and serotypes and to identify virulence, plasmid content, antibiotic resistance and sanitizer tolerance genes using whole genome sequencing.

## 2 Materials and Methods

### 2.1 Sample Collection and Description

All samples included in this study were received from a South African National Accreditation System (SANAS) food laboratory (Microchem Lab Services (Pty) Ltd) in the Western Cape, South Africa. A sample set of 60 isolates was selected from many received over 4 years (2018–2021), originating from various foods and food‐processing environments. Samples were grouped into three categories, namely RTE hummus (*n* = 21), fresh produce (*n* = 8) and the food‐processing environment (*n* = 31). The samples from the food‐processing environment were not necessarily associated with the factories from which the hummus and fresh produce originated. Food‐processing environment samples were obtained from floors (*n* = 7), drains (*n* = 14), food contact surfaces (*n* = 3), equipment (*n* = 2), cleaning tools (*n* = 2), chiller door handles (*n* = 1) and a worker's hand (*n* = 1) and boots (*n* = 1). Fresh produce samples included potato (*n* = 1), cucumber (*n* = 2), coriander/cilantro (*n* = 1), cling peach (*n* = 1), leek (*n* = 1), spinach (*n* = 1), and unspecified vegetables (prewash) (*n* = 1). RTE hummus varieties ranged from an original or unspecified variety (*n* = 11) to jalapeno (*n* = 3), Za'atar (*n* = 1), orange (*n* = 2), peri‐peri (*n* = 1), red pepper (*n* = 1) and coriander chilli (*n* = 2). All isolates were received on RAPID'Lmono chromogenic agar and stored at −4°C.

### 2.2 Detection of Presumptive Positive *L. monocytogenes*

To ensure isolate viability, presumptive positive *L. monocytogenes* were re‐streaked onto RAPID'Lmono chromogenic agar (AEC Amersham) and incubated at 37°C for 24 h. Colonies exhibiting characteristic *L. monocytogenes* growth (blue in color) were streaked onto 2% blood plates (National Health Laboratory Services (NHLS), Greenpoint) for purity and incubated at 37°C for 24 h. Glycerol stocks of the isolates (from the 2% blood plates) were prepared in skim milk, tryptone, glucose and glycerin (STGG) (NHLS) for storage at −20°C.

### 2.3 DNA Extraction and PCR Confirmation

DNA extraction (of *L. monocytogenes* on the 2% blood plates) was performed using a Quick‐DNA Fungal/Bacterial Miniprep Kit (ZymoResearch, Inqaba Biotec) according to the manufacturer's instructions. UltraPure DNase/RNase‐free distilled water (Thermo Fischer Scientific) replaced the bacterial culture to create the negative extraction control. DNA extracts were stored at −20°C.

PCR amplification of the haemolysin (*hly*) gene genotypically confirmed isolates to be *L. monocytogenes* (Blais et al. 1995; Rip and Gouws 2020). Cycle parameters are described by Rip and Gouws (2020). PCR products were separated and visualized by gel electrophoresis on a 1.5% agarose gel (Lonza, Whitehead Scientific) stained with SmartGlow pre‐stain (Whitehead Scientific). A 100 bp DNA ladder (GeneRuler, Thermo Fischer Scientific) was used as a reference marker and 1X Tris base, acetic acid and EDTA (TAE) as a running buffer in the electrophoresis tank. A Gel Doc XR+ System with Image Lab Software (Bio‐rad) was utilized to view the PCR amplicons. Negative DNA extraction controls, a negative PCR control (UltraPure DNase/RNase‐free distilled water (Thermo Fischer Scientific) used instead of template DNA) and a positive PCR control (*L. monocytogenes* ATCC 23074) were included.

### 2.4 Lineage Typing Using RFLP

Lineage typing was performed using the RFLP method developed by Rip and Gouws (2020) with enzymes *Nde*I, *Bfol*I and *Bsh*12851. The concentrations of the restriction enzymes were slightly modified. The 10 μl (total volume) restriction digest included 0.25 unit/μl FastDigest restriction enzyme (Thermo Fischer Scientific). Undigested *L. monocytogenes* DNA served as a negative control and *L. monocytogenes* ATCC 23074 (serotype 4b), *L. monocytogenes* ATCC 7644 (serotype 1/2c) and *L. monocytogenes* ATCC 19114 (serotype 4a) as positive controls for lineage I, II and III, respectively.

### 2.5 WGS

#### 2.5.1 Subset Selection

A subset of 20 isolates was selected from the 60 that were characterized into lineage groups. Isolates, balanced between lineage I and II, were selected to provide a snapshot of the diversity, resistance, and virulence factors across different sources. The subset comprised of 10 lineage I isolates and 10 lineage II isolates from a range of years (2018–2021). Samples were grouped into three categories: RTE hummus (6), fresh produce (6) and the food‐processing environment (8). Eighteen of the 20 isolates were sequenced by CosmosID (Maryland, United States of America (USA)) and two by the Central Analytical Facilities (CAF), Stellenbosch University (Stellenbosch, South Africa). The latter two samples were added after the initial set was sequenced and were therefore processed by a local service provider.

#### 2.5.2 DNA Quality Control

A Nanodrop Lite spectrophotometer (Thermo Fischer Scientific) was used to determine the purity of all DNA extracts through absorbance measures at 260/280 nm before WGS.

#### 2.5.3 WGS by CosmosID

DNA samples (*n* = 18) were quantified and prepared for WGS following the methodology described by Lambrechts et al. (2024), using the Nextera XT DNA Library Preparation Kit (Illumina) and sequenced on an Illumina HiSeq X platform. Sequence data were processed and assembled with BBDuk (Bushnell 2014), SPAdes (Bankevich et al. 2012), and CheckM (Parks et al. 2015). The assembled contigs were analyzed using the CosmosID core genome SNP typing pipeline to assess phylogenetic placement and SNP variations for epidemiological interpretation. This pipeline employed Parsnp (Treangen et al. 2014) as the core genome aligner, accounting for recombination events and genomic variations during alignment with default parameters. The resulting core‐genome SNP set was used to construct phylogenomic relationships via FastTree2 (Price et al. 2010).

#### 2.5.4 WGS by Central Analytical Facilities (CAF)

DNA libraries (*n* = 2) were prepared using the Ion Plus Fragment Library Kit (Thermo Fisher Scientific) according to the manufacturer's instructions. Genomic DNA was fragmented using the Covaris S2 focused ultrasonicator (Covaris) with a 10% duty cycle, 5% intensity and 200 bursts in one cycle, consisting of a 60‐second treatment. Sheared DNA was purified using 1.8x Agencourt AMPure XP reagent (Beckman Coulter). Fragmented DNA underwent end‐repair at room temperature for 20 min, followed by purification with 1.8x Agencourt AMPure XP reagent. End‐repaired DNA was blunt‐end ligated to IonCode Barcode Adapters at room temperature for 30 min and purified using 1.4x Agencourt AMPure XP reagent. The adapter‐ligated library was size selected on the Pippen Prep (Sage Science) using a 2% dye‐free gel cassette with marker L to retain DNA fragments of 630 bp. The adapter‐ligated, size‐selected library was amplified across eight PCR cycles. The amplified libraries were purified using 1x Agencourt AMPure XP reagent (Beckman Coulter) and analyzed for fragment size distribution on the LabChip GX Touch 24 Nucleic Acid Analyzer with the X‐Mark DNA LabChip and HT DNA NGS 3K reagent kit (PerkinElmer) following the manufacturer's protocol. Library fragments were quantified using the Ion Library TaqMan Quantification kit (Thermo Fisher Scientific). Quantitative PCR was conducted using the StepOnePlus Real‐Time PCR System (Thermo Fisher Scientific), and massively parallel sequencing was carried out on the Ion GeneStudio S5 Prime System.

A de novo genome assembly was generated from the trimmed short reads using Unicycler (v0.5.1) (Wick et al. 2017). The analysis was performed on the Galaxy platform (Afgan et al. 2018) in short‐read‐only mode with default parameters.

### 2.6 Bioinformatic Analysis

#### 2.6.1 Sequence Typing

In silico sequence typing involved submitting FASTQ/A files to the MLST‐2.0 tool from the Centre for Genomic Epidemiology (CGE) and selecting a 5x minimum depth for an allele (https://cge.food.dtu.dk/services/MLST/) (Bartual et al. 2005; Clausen et al. 2018; Griffiths et al. 2010; Jaureguy et al. 2008; Larsen et al. 2012; Lemee et al. 2004; Wirth et al. 2006). Seven housekeeping genes were targeted including *abcZ*, *bglA*, *cat*, *dapE*, *dat*, *ldh* and *lhkA*. Serotypes were then inferred from this data.

#### 2.6.2 The Identification of Virulence Genes

Virulence genes were detected using two bioinformatic tools: VirulenceFinder 2.0 from CGE (https://cge.food.dtu.dk/services/VirulenceFinder/) (Clausen et al. 2018; Joensen et al. 2014; Tetzschner et al. 2020) and the BIGSdb‐Lm database maintained at the Institut Pasteur (https://bigsdb.pasteur.fr/listeria/).

#### 2.6.3 The Identification of Antibiotic Resistance Genes

Acquired antimicrobial resistance genes were identified using the ResFinder 4.1 tool from the CGE with a 90% threshold ID and a 60% minimum length (https://cge.food.dtu.dk/services/ResFinder/) (Bortolaia et al. 2020; Clausen et al. 2018; Zankari et al. 2020).

#### 2.6.4 The Identification of Plasmids

The presence of plasmids was determined by uploading the FASTQ files to the PlasmidFinder 2.1 tool from the CGE with a 95% minimum identity threshold and 60% minimum coverage (https://cge.food.dtu.dk/services/PlasmidFinder/) (Carattoli et al. 2014; Clausen et al. 2018).

#### 2.6.5 The Identification of Sanitizer Tolerance Genes

Genomes were annotated using Prokka v1.14.6 (Seemann 2014) and Bakta v1.8.2. (Schwengers et al. 2021) on the Proksee web server (Grant et al. 2023; https://proksee.ca). Sanitizer tolerance‐associated genes (*bcr*ABC, *qac*H, *emr*C) were identified from the annotation output.

### 2.7 Statistical Analysis

Binomial and Chi‐Square tests for equal proportions were performed to identify significant associations between categories and isolate typing results. A *p*‐value < 0.05 was considered a statistically significant difference. All analyses were performed using Statistica 14.0 software.

### 2.8 Disc Diffusion Method

Phenotypic antibiotic resistance testing was performed using the disc diffusion method according to the standard procedure described by The European Committee on Antimicrobial Susceptibility Testing (EUCAST) (EUCAST 2012). Overnight cultures on nonselective, 2% blood plates (NHLS) were suspended in 3 mL 0.85% saline solution (bioMérieux) and the turbidity was adjusted to a 0.5 McFarland standard using the DENSICHEK (bioMérieux). Within 15 min of preparation, the suspensions were spread evenly over the entire surface of Mueller‐Hinton (MH‐F) agar plates (supplemented with 5% defibrinated horse blood and 20 mg/L *β*‐NAD) (Oxoid, Thermo Fischer Scientific) by swabbing in three different directions using sterile cotton swabs. Antibiotic discs (Thermo Fischer Scientific) were dispensed on the surface of the inoculated plates and the plates were incubated at 37°C for 16–20 h.

Seven clinically relevant antibiotics were analyzed in this study: ampicillin (2 micrograms (μg)), chloramphenicol (30 μg), co‐trimoxazole (25 μg), erythromycin (15 μg), gentamicin (10 μg), meropenem (10 μg) and tetracycline (30 μg). A ruler was used to measure the zones of inhibition to the nearest millimeter (mm). Isolates were classified as resistant or susceptible to each antibiotic according to EUCAST (2022) criteria. Breakpoints for chloramphenicol, gentamicin and tetracycline have not yet been established for *L. monocytogenes*, therefore the breakpoints for *Staphylococcus aureus* were used to interpret these results (Andriyanov et al. 2021; Maćkiw et al. 2016; Noll et al. 2018). Testing was performed in duplicate. The control strain used was *S. aureus* ATCC 25923.

## 3 Results and Discussion

### 3.1 PCR and Lineage Typing

*L. monocytogenes* can be classified below the species level into four lineage groups and 13 common serotypes (Maury et al. 2016). These serotypes can further be divided into STs (Maury et al. 2016). Different subtypes (lineage types, serotypes and STs) of the bacteria are associated with varying degrees of virulence and different clinical outcomes (Bouymajane et al. 2021; Lotfollahi et al. 2017; Orsi et al. 2011). PCR results (*hly* gene presence) confirmed all phenotypic presumptive positive isolates to be *L. mono*cytogenes. Of the 60 isolates lineage typed, 56.7% were categorized as lineage I and 43.3% were lineage II (*p* = 0.12) (Table S1). The overrepresentation of lineage I *L. monocytogenes* in the South African food industry is aligned with other South African studies (Keet and Rip 2021; Matle et al. 2020b; Rip and Gouws 2020).

For RTE hummus (*n* = 21), 47.6% of isolates were determined to be lineage I and 52.4% to be lineage II. For fresh produce (*n* = 8), 87.5% of isolates were found to be lineage I and 12.5% to be lineage II. With regard to the food‐processing environment (*n* = 31), 54.8% of isolates were classified as lineage I and 45.2% as lineage II. At an *α* level of 5%, samples for RTE hummus (*p* = 0.33) and the food‐processing environment (*p* = 0.36) did not provide sufficient evidence to conclude that the proportions of lineage I and lineage II were significantly different. However, lineage I was significantly associated with fresh produce (*p* = 0.04). For this category, all isolates but one were found to be lineage I. A similar South African study by Kayode and Okoh (2022) also observed a prevalence of lineage I in fresh produce in the Eastern Cape province: all 90 isolates analyzed were characterized as lineage I. Fresh produce may become contaminated during cultivation or postharvest handling and distribution (Cordano and Jacquet 2009; Matereke and Okoh 2020; Townsend et al. 2021; Zhu et al. 2017). A review study detected *L. monocytogenes* in each stage of the fresh produce supply chain with the greatest prevalence in natural environments (Townsend et al. 2021). Irrigation water (Iwu and Okoh 2020), manure (Gholipour et al. 2020) and agricultural soil (Townsend et al. 2021) have been reported as reservoirs and potential transmission routes of *L. monocytogenes* contamination. The continued recovery of lineage I from fresh produce suggests this lineage type to be well adapted to proliferate within these foods and their cultivation environments.

### 3.2 Sequence Typing and Sanitizer Tolerance Genes

**Figure 1:**
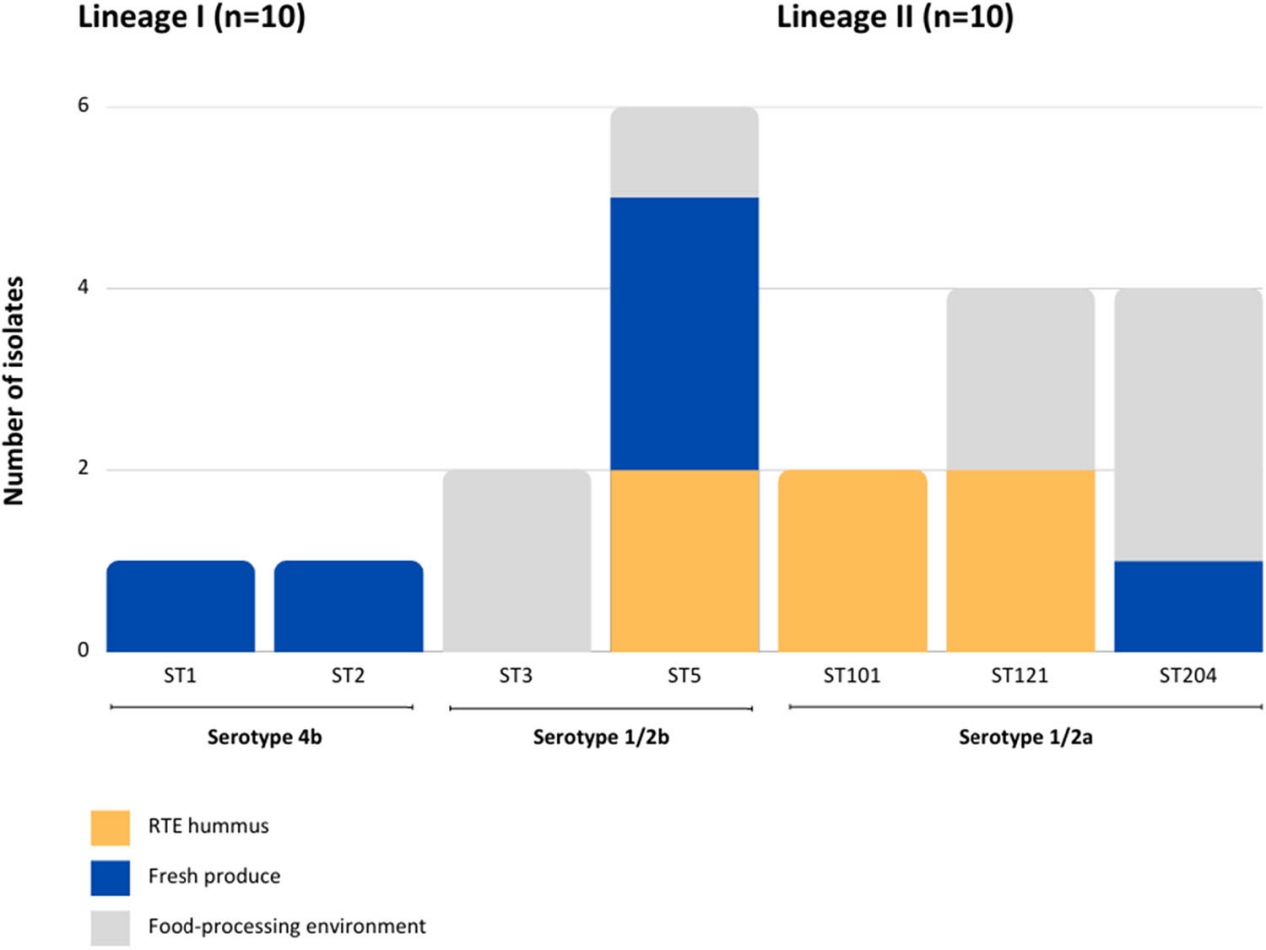
The distribution of STs for 20 *L. monocytogenes* isolates from RTE hummus, fresh produce and the food‐processing environment in the Western Cape, South Africa.

The 20 isolates sequenced were found to be of seven different STs: ST5 (30%; *n* = 6), ST121 (20%; *n* = 4), ST204 (20%; *n* = 4), ST3 (10%; *n* = 2), ST101 (10%; *n* = 2), ST1 (5%; *n* = 1) and ST2 (5%; *n* = 1) (*p* = 0.30) (Figure 1). Clinical isolates analyzed during the South African outbreak were represented by nine STs (NICD 2018), including ST1, ST2, ST5 and ST101 (reported in this current study), however the genetic relatedness of these isolates is unknown. The genetic diversity seen in this study suggests that several sources could be linked to the contamination of these food and environmental categories.

For the food‐processing environment (*n* = 8) (Figure 1), ST204 (37.5%), ST121 (25%), ST3 (25%) and ST5 (12.5%) were recovered. ST3 (serotype 1/2b) was limited to this category. Regarding fresh produce (*n* = 6), ST5 (50%), ST204 (16.7%), ST1 (16.7%) and ST2 (16.7%; *n* = 1) were identified. ST1 and ST2 (both serotype 4b) were only isolated from fresh produce. For RTE hummus (*n* = 6), equal proportions of ST5 (33.3%), ST101 (33.3%) and ST121 (33.3%) were recovered. ST101 (serotype 1/2a) was only associated with this category. At an *α* level of 5%, the samples for RTE hummus (*p* = 1.00), fresh produce (*p* = 0.73) and the food‐processing environment (*p* = 0.96) did not provide sufficient evidence to conclude that any one ST was significantly associated with a category. This is primarily due to a lack of power of the small sample sizes. Many studies report ST1, ST2, ST3, ST6 and ST101 to cause human infection (Filipello et al. 2020; Kremer et al. 2017; Mafuna et al. 2021; Mammina et al. 2013).

Factory origin information of the isolates was requested from the supplier after analysis for discussion purposes (Table 1; Table S1). Contamination of RTE hummus either originates from raw ingredients or, more commonly, from cross‐contamination within the food processing environment (Garner and Kathariou 2016; Jordan et al. 2018; Rugna et al. 2021; Salazar et al. 2020). It was observed that ST5 (serotype 1/2b) was recovered from a hummus blender, two hummus samples (original and coriander chilli varieties) and coriander (fresh produce) in a RTE and deli food factory (Factory J) in 2019 (Table S2). This finding suggests possible cross‐contamination within this facility and emphasizes the importance of good manufacturing practices, appropriate factory design and production flow, correct storage of raw ingredients and RTE products, and effective cleaning and sanitation. Based on phylogenetic analysis (SNP tree), there were no branch distance between these ST5 isolates and the ST5 isolates from Factory K (spinach and vegetable prewash in 2018 and 2019 respectively) (Figure S1).

**Table 1:** LIPI‐1, LIPI‐2 and LIPI‐3 virulence genes and sanitizer tolerance genes identified in 20 *L. monocytogenes* isolates from RTE hummus, fresh produce and the food‐processing environment in the Western Cape, South Africa.

Category	Isolate number corresponds to WGS file number	Sample description	Year	Subtype (lineage type, serotype, ST)	Factory origin	Virulence genes		Sanitizer tolerance genes±		
						LIPI‐1 and LIPI‐2	LIPI‐3	*bcr*ABC	*emr*C	*qac*H
Food‐processing environment	3	Boots	2019	I, 1/2b, ST3	(C) Pie factory	+	+		+	
	6	Floor (after cleaning)	2021	I, 1/2b, ST3	(G) Uncooked and RTE meat factory	+	+		+	
	2	Equipment (hummus blender)	2019	I, 1/2b, ST5	(J) RTE and deli food factory	+		+		
	1	Surface (cutting board)	2018	II, 1/2a, ST121	(G) Uncooked and RTE meat factory	+				+
	7	Chiller door and handles	2021	II, 1/2a, ST121	(G) Uncooked and RTE meat factory	+				+
	19^a^	Worker's hand (during production)	2018	II, 1/2a, ST204	(G) Uncooked and RTE meat factory	+		+		
	4	Drain (hot chicken)	2020	II, 1/2a, ST204	(J) RTE and deli food factory	+		+		
	5	Floor (dispatch area)	2020	II, 1/2a, ST204	(G) Uncooked and RTE meat factory	+		+		
Fresh produce	13	Leeks	2021	I, 4b, ST1	(J) RTE and deli food factory	+	+			
	9	Potato	2018	I, 4b, ST2	(K) RTE airline food factory	+				+
	8	Spinach	2018	I, 1/2b, ST5	(K) RTE airline food factory	+		+		
	11	Coriander/cilantro	2019	I, 1/2b, ST5	(J) RTE and deli food factory	+		+		
	12	Vegetable (prewash)	2019	I, 1/2b, ST5	(K) RTE airline food factory	+		+		
	10	Cucumber	2019	II, 1/2a, ST204	(J) RTE and deli food factory	+		+		
RTE hummus	15	Hummus	2019	I, 1/2b, ST5	(J) RTE and deli food factory	+		+		
	14	Hummus (coriander chilli)	2019	I, 1/2b, ST5	(J) RTE and deli food factory	+		+		
	17	Smoked hummus	2021	II, 1/2a, ST101	(J) RTE and deli food factory	+		+		
	18	Smoked hummus	2021	II, 1/2a, ST101	(J) RTE and deli food factory	+		+		
	20^a^	Hummus	2018	II, 1/2a, ST121	(J) RTE and deli food factory	+				+
	16	Hummus (orange)	2019	II, 1/2a, ST121	(J) RTE and deli food factory	+				+

*Note:* Factory origins of the same letter (C, G, J, K) represent the same factory.

+ Gene present.

^±^ Sanitizer tolerance genes were identified using the Proksee web server (Grant et al. 2023; https://proksee.ca/) with tools Prokka (Seemann 2014) and Bakta (Schwengers et al. 2021).

^a^ The two isolates underwent WGS by Central Analytical Facilities (Stellenbosch University). The remaining isolates were sequenced by Cosmos ID. Due to the use of different platforms and service providers, the resulting assemblies were not directly comparable. Nonetheless, this variation did not affect the study's outcomes, as the focus remained on the targeted genes relevant to the research objectives.

Quaternary ammonium compounds (QACs) are frequently employed as disinfectants in food processing plants (Müller et al. 2014). The expression of genes in *L. monocytogenes* that confer tolerance to QACs can complicate sanitation efforts, potentially leading to the organism's survival if effective control measures are not implemented. This survival heightens the risk of human exposure to the pathogen. Tolerance to certain QACs, such as benzalkonium chloride (BC), is linked to the presence of genes that code for efflux pumps, including *brc*ABC, *qac*H, and *emr*C. These genes lead to higher minimum inhibitory concentrations for BC, allowing the bacteria to survive at concentrations that would otherwise be lethal (Kragh et al. 2024).

ST2 and ST5 strains recovered from the RTE airline food factory (Table 1) were introduced with fresh produce (potato and spinach) and may likely cross‐contaminate RTE foods produced in this facility, which is concerning. These isolates contained the *qac*H and *bcr*ABC gene cassette respectively.

**Figure 2:**
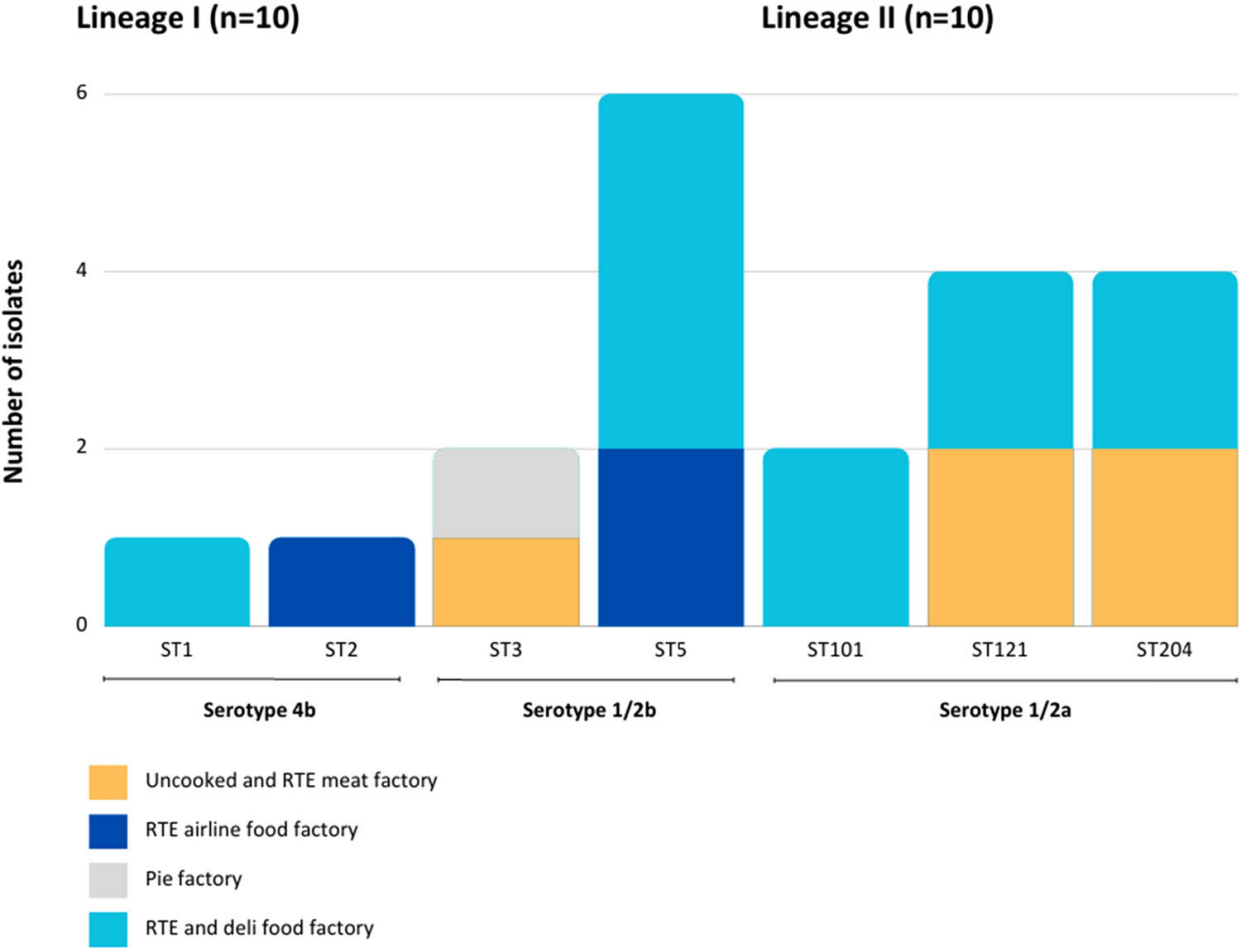
The distribution of STs for 20 *L. monocytogenes* isolates from an uncooked and RTE meat factory, a RTE airline food factory, a pie factory and a RTE and deli food factory.

It was noted that three of the four factory origins (G, J and K) supported the survival of more than one *L. monocytogenes* strain, suggesting the coexistence of subtypes within facilities (Table 1). It was also evident that ST3, ST5, ST121 and ST204 were able to proliferate in processing environments manufacturing very different foods (Figure 2). An ST3 isolate was recovered from a floor post‐cleaning (Table 1). The *emr*C gene was detected in both ST3 environmental isolates (together with LIPI‐3 virulence genes). If cleaning protocols are ineffective, the *emr*C gene may enable the bacteria to survive the sanitizer application step. Further, the *bcr*ABC gene was limited to ST5 isolates across the food and environment categories, ST204 and ST101 (Table 1).

Repetitive recovery of ST121 and ST204 in a RTE and deli food factory (Factory J) and in an uncooked and RTE meat processing facility (Factory G) over the years questioned whether these strains might be persisting in these environments (Figure 2). The phylogenetic analysis (SNP tree) revealed no branch distance among the ST121 isolates or the ST204 isolates out of the 18 isolates included in Figure S1 (isolate details in Table 1). Schmitz‐Esser et al. (2015) confirmed ST121 to be pervasive in food‐processing facilities and analyzed ST121 strains that persisted for 8 years in production plants in Ireland and Austria. A South African study by Mafuna et al. (2021) identified various stress tolerance genes, benzalkonium chloride tolerance genes and biofilm formation genes in ST204 and reported this ST (as well as others) to be well‐adapted to the food‐processing environment. Three ST121 isolates in this current study (two from the environment and one from hummus) carried the *qac*H gene, conferring tolerance to benzalkonium chloride.

### 3.3 Serotyping

The seven STs identified belonged to three serotypes: serotype 1/2a (50%; *n* = 10), 1/2b (40%; *n * = 8) and 4b (10%; *n* = 2) (*p* = 0.07) (Figure 1). Serotypes 1/2a, 1/2b and 4b are collectively responsible for 95% of all described human listeriosis cases, representing a significant public health threat in South Africa (Bouymajane et al. 2021; Lotfollahi et al. 2017). These serotypes are evidently able to overcome the stresses implicated in the South African food industry and exhibit a competitive advantage over other serotypes.

For the food‐processing environment (*n* = 8), serotypes 1/2a (62.5%) and 1/2b (37.5%) were recovered. For fresh produce (*n* = 6), serotypes 1/2b (50%), 4b (33.3%) and 1/2a (16.7%) were identified. Serotype 4b was only found in fresh produce. For RTE hummus (*n* = 6), serotypes 1/2a (66.7%) and 1/2b (33.3%) were isolated. No single serotype was significantly over‐represented in any category (RTE hummus (*p* = 0.69), fresh produce (*p* = 0.88) or the food‐processing environment (*p* = 0.73)).

Serotype 4b (isolated from potato and leeks in different facilities) is considered the most pathogenic serotype (Todd and Notermans 2011; Yu and Jiang 2014). This serotype has previously been recovered from various fruits and vegetables in many different countries and has caused listeriosis outbreaks associated with RTE salads, caramel apples, stone fruit, bean sprouts and leafy greens in the last 10 years (CDC 2015a; CDC 2015b; CDC 2016; Garner and Kathariou 2016; Stephan et al. 2015).

### 3.4 Antibiotic Resistance

Genotypic analysis found all 20 isolates to contain the *fosX* gene, encoding resistance to fosfomycin (Table S2). *L. monocytogenes* is intrinsically resistant to fosfomycin (Noll et al. 2018; Parra‐Flores et al. 2022; Troxler et al. 2000), meaning that inherent characteristics of the bacteria promote natural resistance to the antibiotic (Olaimat et al. 2018b). Two lineage I, serotype 1/2b, ST5 isolates from RTE hummus also contained the *tetM* gene, encoding resistance to tetracycline, doxycycline and minocycline (Table S2). This finding was consistent with phenotypic disc diffusion results that showed resistance to tetracycline. Resistance of *L. monocytogenes* from RTE hummus to these antibiotics has not yet been reported. Only one study analyzed phenotypic antibiotic susceptibility of *L. monocytogenes* isolates from RTE hummus (*n* = 15) and reported resistance to chloramphenicol (*n* = 1) and erythromycin (*n* = 2) only (Keet and Rip 2021). Disc diffusion method results were highly concordant with those of WGS (Table S2), except that a ST2 isolate from potato also showed resistance to co‐trimoxazole. This resistance finding is problematic (especially for penicillin‐sensitive people) as this strain is repeatedly associated with foodborne outbreaks. Phenotypic antibiotic susceptibility testing revealed susceptibility to ampicillin (100%), chloramphenicol (100%), erythromycin (100%), meropenem (100%), and gentamicin (100%). Overall, isolates were highly susceptible to all main antibiotics prescribed to treat listeriosis. However, other publications have illustrated that resistance patterns can change over time and so continued surveillance is important.

### 3.5 Virulence

Various virulence factors contribute to the pathogenicity of *L. monocytogenes* by allowing it to interact with and survive the killing mechanisms of host cells and spread from one infected cell to others (Kathariou 2002; Matle et al. 2020a). The most important virulence determinants are clustered together to comprise LIPI‐1 (*prfA, plcA, hly, mpl, actA, plcB*) and LIPI‐2 (*inlA, inlB, inlC, inlJ*) (Anwar et al. 2022; Matle et al. 2020a). These gene clusters coordinate the main steps of infection and therefore determine *L. monocytogenes* pathogenicity (Kayode and Okoh 2022). LIPI‐3 (*llsA, llsG, llsH, llsX, llsB, llsY, llsD, llsP)* (which encodes Listeriolysin S (LLS), a hemolytic and cytotoxic factor) is most often confined to lineage I, can alter the host microbiota, and is associated with enhanced virulence (Guidi et al. 2021; Smith et al. 2019b). In this study, isolates were screened for all genes comprising LIPI‐1, LIPI‐2, LIPI‐3 and LIPI‐4 (VirulenceFinder 2.0 from CGE and BIGSdb‐Lm (Institut Pasteur)). No genes comprising LIPI‐4 (*licG, licB, licA, lm900558‐70012, lm900558‐70013*) were identified in any of the isolates. Complete LIPI‐1 and LIPI‐2 gene clusters, critical to the infectious cycle of *L. monocytogenes*, were detected in all isolates and have the potential to be expressed in the human host. Additionally, two isolates (ST3 from the food‐processing environment and ST1 from fresh produce) carried the LIPI‐3 gene cluster (Table 1).

It was noted that various other virulence genes (Table S2) detected in all isolates, when expressed, may promote stress tolerance, which could explain why these particular strains are difficult to eradicate from food processing facilities. Based on WGS analysis, a total of 91 virulence genes were detected in the 20 *L. monocytogenes* isolates. The basic functions of a few of these genes are presented in Table S3. The *sigB* gene has been associated with the survival of *L. monocytogenes* in unfavorable conditions and is connected to the resistance of biofilms to sanitation agents (Unrath et al. 2021; van der Veen and Abee 2010). The *degU* gene is required for growth at high temperatures and adherence to plastic surfaces (Unrath et al. 2021). The *sigB, degU, AgrA* and *flaA* genes all mediate the formation of biofilms, which increases the ability of bacteria to adhere to surfaces (Unrath et al. 2021; van der Veen and Abee 2010). Bacteria enclosed within biofilms are protected from desiccation, nutrient deprivation and disinfection procedures (Unrath et al. 2021). However, further research is needed to understand the process and mechanisms by which *L. monocytogenes* adapt to these foods and their different processing environments.

### 3.6 Plasmids

Survival in unfavorable conditions can also be linked to plasmid harborage. Since plasmids often carry genes that contribute to stress response and environmental adaptation, the identification of these extrachromosomal mobile genetic elements provides some explanation for why certain strains persist over others (Gray et al. 2021; Naditz et al. 2019; Schmitz‐Esser et al. 2021; Schmitz‐Esser et al. 2015). It has been suggested that certain lineage types, serotypes and STs are more likely to carry plasmids than others. Schmitz‐Esser et al. (2021) found 45% of lineage I strains and 61% of lineage II strains to contain plasmids. High proportion of ST121 (92%), ST5 (88%), ST8 (79%), ST3 (78%) and ST204 (71%) strains carried plasmids (Schmitz‐Esser et al. 2021). A low prevalence of plasmids (less than 15%) was seen in ST4, ST1 and ST2 strains (Schmitz‐Esser et al. 2021).

In this study, all isolates except two (ST1 and ST2) contained one plasmid (Table S2). These were recovered from different factory origins in different years. Two different plasmid groups were identified: Plasmid incompatibility groups (Inc)18 (rep25) and Inc18 (rep26). Seven isolates (ST3, ST5, ST101) carried Inc18(Rep25) and 11 (ST5, ST121, ST204) carried Inc18(Rep26). Both plasmids have previously been identified in *L. monocytogenes* (Parra‐Flores et al. 2022).

## 4 Conclusion

The data generated from this study provide some insight into the diversity of *L. monocytogenes* in the categories of interest. This type of genetic profiling is very limited for *L. monocytogenes* in South Africa, especially for RTE hummus (underreported globally) and fresh produce. Lineage I *L. monocytogenes* seems to persist in the South African food industry for reasons that need to be further explored. The repetitive recovery of lineage I from fresh produce suggests that this lineage type is well adapted to proliferate within these foods and their cultivation environments.

The identification of full LIPI‐1 and LIPI‐2 gene clusters in the large majority of isolates confirms the potential of these subtypes to cause human infection. The identification of all LIPI‐3 genes in a food‐processing environment isolate (ST3) and a fresh produce isolate (ST1) suggests that these strains are particularly virulent and worth monitoring. All isolates contained the *sigB, degU, agrA, flaA* and *actA* genes suggested to have both virulence and stress tolerance function. Some isolates carried sanitizer tolerance genes *emr*C (ST3), *bcr*ABC (ST5, ST101, ST204) and *qac*H (ST2, ST121) which may impact sanitation efforts. Sequence type 3 isolates (with *emr*C and LIPI‐3) pose a greater health risk if allowed to persist, especially since they were detected on boots and floors after cleaning, highlighting shortcomings in sanitation measures. Both genotypic and phenotypic resistance results showed susceptibility to all clinically relevant antibiotics, indicating their continued effectiveness in treating *L. monocytogenes* infections.

Characterization of *L. monocytogenes* at every stage in the fresh produce supply chain, including cultivation environments, would assist with identifying the source of contamination and help control *L. monocytogenes* in fresh produce. The ubiquity of *L. monocytogenes* in nature makes the contamination of fresh produce more difficult to avoid. Among isolates originating from fresh produce, ST1 and ST2 (linked to outbreaks and severe clinical outcomes in literature), ST5, and ST204 were detected. Washing procedures of fruits and vegetables are further encouraged, especially those consumed raw, along with exploring more effective methods of decontamination.

All hummus samples included in this study originated from the same food factory where ST5, ST101 and ST121 were detected. Sequence type 5 was recovered from hummus, its processing blender, and coriander within this RTE and deli food factory. Phylogenetic analysis showed that these isolates were indistinguishable from ST5 strains identified in a separate RTE airline food factory, suggesting a possible shared ingredient source. Factory design and production flow, storage of raw and RTE products, and cleaning and sanitation of this facility urgently need to be improved to mitigate cross‐contamination of RTE hummus. Potentially persistent strains residing in this facility represent a source of recurrent contamination.

## Author Contributions

**Samantha Anne du Toit:** investigation, writing – original draft (lead), review and editing, visualization (lead), formal analysis, methodology (lead). **Diane Rip:** supervision, funding acquisition, conceptualization (lead), investigation, writing, review and editing (equal), formal analysis (supporting), methodology (supporting). **Pieter Gouws:** supervision, review (final draft).

## Ethics Statement

This study study was approved by the Research Ethics Committee at Stellenbosch University: Biological and Environmental Safety #19029.

## Conflicts of Interest

The authors declare no conflicts of interest.

## Supporting information


**Supporting Figure 1:** SNP tree based on core genome phylogeny for *L. monocytogenes* isolates 1‐18 (highlighted in blue) generated by CosmosID, Maryland. Isolate numbers in blue (1‐18) correspond to descriptions provided in Table 1. Black text are reference genomes.

**Supporting Table 1:** Lineage typing results for 60 *L. monocytogenes* isolates from RTE hummus, fresh produce and the food‐processing environment in the Western Cape, South Africa.

**Supporting Table 2:** WGS characterisation, resistance gene profiling and virulence gene profiling results for 20 *L. monocytogenes* isolates from RTE hummus, fresh produce and the food‐processing environment in the Western Cape, South Africa.

**Supporting Table 3:** The basic functions of virulence genes detected in *L. monocytogenes* isolates in this study (Dussurget 2008).

## Data Availability

The data that support the findings of this study are openly available in GenBank at https://www.ncbi.nlm.nih.gov/bioproject/, reference number PRJNA1298896, and additional data supporting the findings are included in the supporting material of this article.
