# Supplementary material for: Profiling Listeria monocytogenes in Hummus, Fresh Produce, and Food Processing Environments in the Western Cape, South Africa
Source: Microbiologyopen. 2025 Sep 8;14(5):e70060. doi: 10.1002/mbo3.70060 (PMC12417568; doi:10.1002/mbo3.70060)
Supplement: Supplementary file 1 — Supporting Figure 1: SNP tree based on core genome phylogeny for L. monocytogenes isolates 1‐18 (highlighted in blue) generated by CosmosID, Maryland. Isolate numbers in blue (1‐18) correspond to descriptions provided in Table 1. Black text are reference genomes. [file MBO3-14-e70060-s003.docx]

**Supplementary Figure 1**: SNP tree based on core genome phylogeny for *L. monocytogenes* isolates 1-18 (highlighted in blue) generated by CosmosID, Maryland. Isolate numbers in blue (1-18) correspond to descriptions provided in Table 1. Black text are reference genomes.
