# Supplementary material for: Profiling Listeria monocytogenes in Hummus, Fresh Produce, and Food Processing Environments in the Western Cape, South Africa
Source: Microbiologyopen. 2025 Sep 8;14(5):e70060. doi: 10.1002/mbo3.70060 (PMC12417568; doi:10.1002/mbo3.70060)
Supplement: Supplementary file 2 — Supporting Table 1: Lineage typing results for 60 L. monocytogenes isolates from RTE hummus, fresh produce and the food‐processing environment in the Western Cape, South Africa. [file MBO3-14-e70060-s002.docx]

**Supplementary Table 1** Lineage typing results for 60 *L. monocytogenes* isolates from RTE hummus, fresh produce and the food-processing environment in the Western Cape, South Africa

| **Category** | **Sample description** | **Year** | **Factory origin** | **Lineage Type** |
| --- | --- | --- | --- | --- |
| **Food-processing environment** | Drain (preparation area) | 2018 | (A) Condiment factory | I |
|  | Drain (production area) | 2018 | (A) Condiment factory | I |
|  | Drain (mixer) | 2018 | (B) Bread factory | I |
|  | Boots | 2019 | (C) Pie factory | I |
|  | Drain (preparation area) | 2019 | (A) Condiment factory | I |
|  | Drain | 2019 | (D) Commercial bakery | I |
|  | Drain | 2019 | (D) Commercial bakery | I |
|  | Equipment (hummus blender) | 2019 | (J) RTE and deli food factory | I |
|  | Drain (mixer) | 2020 | (B) Bread factory | I |
|  | Drain | 2020 | (E) Sanitizing product producer | I |
|  | Floor (juice holding room) | 2020 | (F) Dairy | I |
|  | Floor (cooler) | 2020 | (B) Bread factory | I |
|  | Drain | 2021 | (B) Bread factory | I |
|  | Floor (after cleaning) | 2021 | (G) Uncooked and RTE meat factory | I |
|  | Floor (packing) | 2021 | (H) Snack and wrap factory | I |
|  | Floor (flour store) | 2021 | (H) Snack and wrap factory | I |
|  | Crate wash machine | 2021 | (B) Bread factory | I |
|  | Worker’s hand (during production) | 2018 | (G) Uncooked and RTE meat factory | II |
|  | Drain (cooking area) | 2018 | Unknown | II |
|  | Surface (cutting board) | 2018 | (G) Uncooked and RTE meat factory | II |
|  | Surface (production table) | 2018 | (I) Pork deli meat factory | II |
|  | Floor | 2018 | (G) Uncooked and RTE meat factory | II |
|  | Surface (production table) | 2019 | (I) Pork deli meat factory | II |
|  | Equipment (hummus glass) | 2019 | (J) RTE and deli food factory | II |
|  | Drain (hot chicken) | 2020 | (J) RTE and deli food factory | II |
|  | Drain (emulsion) | 2020 | (I) Pork deli meat factory | II |
|  | Drain | 2020 | (G) Uncooked and RTE meat factory | II |
|  | Floor (dispatch area) | 2020 | (G) Uncooked and RTE meat factory | II |
|  | Drain | 2021 | (J) RTE and deli food factory | II |
|  | Chiller door and handles | 2021 | (G) Uncooked and RTE meat factory | II |
|  | Cleaning tool (brushware) | 2021 | (G) Uncooked and RTE meat factory | II |
| **Fresh produce** | Potato | 2018 | (K) RTE airline food factory | I |
|  | Spinach | 2018 | (K) RTE airline food factory | I |
|  | Cucumber | 2019 | (J) RTE and deli food factory | I |
|  | Coriander | 2019 | (J) RTE and deli food factory | I |
|  | Cling peach | 2019 | (L) Ice cream factory | I |
|  | Vegetable (pre-wash) | 2019 | (K) RTE airline food factory | I |
|  | Leeks | 2021 | (K) RTE airline food factory | I |
|  | Cucumber | 2019 | (J) RTE and deli food factory | II |
| **RTE hummus** | Hummus | 2018 | (J) RTE and deli food factory | I |
|  | Hummus | 2018 | (J) RTE and deli food factory | I |
|  | Hummus | 2018 | (J) RTE and deli food factory | I |
|  | Hummus (orange) | 2018 | (J) RTE and deli food factory | I |
|  | Hummus (peri-peri) | 2018 | (J) RTE and deli food factory | I |
|  | Hummus | 2019 | (J) RTE and deli food factory | I |
|  | Hummus | 2019 | (J) RTE and deli food factory | I |
|  | Hummus (classic) | 2019 | (J) RTE and deli food factory | I |
|  | Hummus (coriander chilli) | 2019 | (J) RTE and deli food factory | I |
|  | Hummus (coriander chilli) | 2019 | (J) RTE and deli food factory | I |
|  | Hummus | 2018 | (J) RTE and deli food factory | II |
|  | Hummus (jalapeno) | 2018 | (J) RTE and deli food factory | II |
|  | Hummus (jalapeno) | 2018 | (J) RTE and deli food factory | II |
|  | Hummus (Za’atar) | 2018 | (J) RTE and deli food factory | II |
|  | Hummus (red pepper) | 2018 | (J) RTE and deli food factory | II |
|  | Hummus | 2019 | (J) RTE and deli food factory | II |
|  | Hummus | 2019 | (J) RTE and deli food factory | II |
|  | Hummus (jalapeno) | 2019 | (J) RTE and deli food factory | II |
|  | Hummus (orange) | 2019 | (J) RTE and deli food factory | II |
|  | Smoked hummus | 2021 | (J) RTE and deli food factory | II |
|  | Smoked hummus | 2021 | (J) RTE and deli food factory | II |

Factory origin information was requested from Microchem Lab Services (Pty) Ltd (maintaining anonymity) after lineage typing for discussion purposes.

Factory origins represented by more than one isolate have been colour coded.

No duplicates were added to the sample set.
