# Supplementary material for: Profiling Listeria monocytogenes in Hummus, Fresh Produce, and Food Processing Environments in the Western Cape, South Africa
Source: Microbiologyopen. 2025 Sep 8;14(5):e70060. doi: 10.1002/mbo3.70060 (PMC12417568; doi:10.1002/mbo3.70060)
Supplement: Supplementary file 3 — Supporting Table 2: WGS characterisation, resistance gene profiling and virulence gene profiling results for 20 L. monocytogenes isolates from RTE hummus, fresh produce and the food‐processing environment in the Western Cape, South Africa. [file MBO3-14-e70060-s004.docx]

**Supplementary Table 2** WGS characterisation, resistance gene profiling and virulence gene profiling results for 20 *L. monocytogenes* isolates from RTE hummus, fresh produce and the food-processing environment in the Western Cape, South Africa

| **Category** | **Sample description** | **Year** | **Factory origin** | **Lineage Type** | **Sequence Type** | **Serotype** | **Antibiotic resistance genes** | **Virulence genes^*^** | **Plasmids** |
| --- | --- | --- | --- | --- | --- | --- | --- | --- | --- |
| **Food-processing environment** | Boots | 2019 | (C) Pie factory | I | 3 | 1/2b | *fosX* | *agrA, ami, aut, eut_operon, flaA, gadA, gadB, gadC, gmar, lap, oatA, oppA, orfX, orfZ, rli55, rli60, rsbv, biLE, bsh, btlB, chiA, clpc, clpp, codY, ctaP, ctsR, degU, dltA, fri, fur, gtcA, hfq, hly, htrA, hupC, iap, inlA, inlB, inlC, inlJ, lgt, lhrC, lipA, lisK, lisR, lntA, lpeA, lsp, mogR, mpl, mprf, murA, perR, pgdA, pgl, plcA, plcB, prfA, prsA2, pycA, relA, secA2, sigB, sipX, sipZ, sod, srtA, srtB, stp, svpA, tcsA, tig, uHpt, virR*  (74 genes) | Inc18  Rep25 |
|  | Floor (after cleaning) | 2021 | (G) Uncooked and RTE meat factory | I | 3 | 1/2b | *fosX* | *agrA, ami, aut, eut_operon, flaA, gadA, gadB, gadC, gmar, lap, oatA, oppA, orfX, orfZ, rli55, rli60, rsbv, biLE, bsh, btlB, chiA, clpc, clpp, codY, ctaP, ctsR, degU, dltA, fri, fur, gtcA, hfq, hly, htrA, hupC, iap, inlA, inlB, inlC, inlJ, lgt, lhrC, lipA, lisK, lisR, lntA, lpeA, lsp, mogR, mpl, mprf, murA, perR, pgdA, pgl, plcA, plcB, prfA, prsA2, pycA, relA, secA2, sigB, sipX, sipZ, sod, srtA, srtB, stp, svpA, tcsA, tig, uHpt, virR*  (74 genes) | Inc18  Rep25 |
|  | Equipment (hummus blender) | 2019 | (J) RTE and deli food factory | I | 5 | 1/2b | *fosX* | *agrA, ami, aut, eut_operon, flaA, flgC, gadA, gadB, gadC, gmar, lap, oatA, oppA, orfX, orfZ, rli55, rli60, rsbv, biLE, bsh, btlB, chiA, clpc, clpp, codY, ctaP, ctsR, degU, dltA, fri, fur, gtcA, hfq, hly, htrA, hupC, iap, inlA, inlB, inlC, inlJ, lgt, lhrC, lipA, lisK, lisR, lpeA, lsp, mogR, mpl, mprf, murA, perR, pgdA, pgl, plcA, plcB, prfA, prsA2, pycA, relA, secA2, sigB, sipX, sipZ, sod, srtA, srtB, stp, svpA, tcsA, tig, uHpt, virR*  (74 genes) | Inc18  Rep25 |
|  | Surface (cutting board) | 2018 | (G) Uncooked and RTE meat factory | II | 121 | 1/2a | *fosX* | *actA, agrA, ami, aut, eut_operon, flaA, flgC, flgE, gadB, gadC, lap, lapB, oatA, oppA, orfX, orfZ, rli55, rli60, rsbv, biLE, bsh, btIB, chiA, clpB, clpc, clpe, clpp, codY, ctaP, ctsR, degU, dltA, fbpA, fri, fur, gtcA, hfq, hly, htrA, hupC, iap, inlA, inlB, inlC, inlJ, inlk, lgt, lhrC, lipA, lisK, lisR, lmo0514, lmo0610, lmo2085, lntA, lpeA, lplA1, lsp, mogR, mpl, mprf, murA, perR, pgdA, pgl, plcA, plcB, prfA, prsA2, pycA, recA, relA, secA2, sigB, sipX, sipZ, sod, srtA, srtB, stp, svpA, tcsA, tig, uHpt, vip, virR*  (86 genes) | Inc18  Rep26 |
|  | Chiller door and handles | 2021 | (G) Uncooked and RTE meat factory | II | 121 | 1/2a | *fosX* | *actA, agrA, ami, aut, eut_operon, flaA, flgC, flgE, gadB, gadC, lap, lapB, oatA, oppA, orfX, orfZ, rli55, rli60, rsbv, biLE, bsh, btlB, chiA, clpB, clpc, clpe, clpp, codY, ctaP, ctsR, degU, dltA, fbpA, fri, fur, gtcA, hfq, hly, htrA, hupC, iap, inlA, inlB, inlC, inlJ, inlk, lgt, lhrC, lipA, lisK, lisR, lmo0514, lmo0610, lmo2085, lntA, lpeA, lplA1, lsp, mogR, mpl, mprf, murA, perR, pgdA, pgl, plcA, plcB, prfA, prsA2, pycA, recA, relA, secA2, sigB, sipX, sipZ, sod, srtA, srtB, stp, svpA, tcsA, tig, uHpt, vip, virR*  (86 genes) | Inc18  Rep26 |
|  | Worker’s hand (during production) | 2018 | (G) Uncooked and RTE meat factory | II | 204 | 1/2a | *fosX* | *actA, agrA, ami, aut, eut_operon, flaA, flgC, flgE, gadA, gadB, gadC, lap, lapB, oatA, oppA, orfX, orfZ, rli55, rli60, rsbv, biLE, bsh, btlB, chiA, clpB, clpc, clpe, clpp, codY, ctaP, ctsR, dal, degU, dltA, fbpA, fri, fur, gtcA, hfq, hly, htrA, hupC, iap, inlA, inlB, inlC, inlF, inlH, inlJ, inlk, lgt, lhrC, lipA, lisK, lisR, lmo0514, lmo0610, lmo2026, lmo2085, lntA, lpeA, lplA1, lsp, mogR, mpl, mprf, murA, perR, pgdA, pgl, plcA, plcB, prfA, prsA2, pycA, recA, relA, secA2, sigB, sipX, sipZ, sod, srtA, srtB, stp, svpA, tcsA, tig, uHpt, virR*  (90 genes) | Inc18  Rep26 |
|  | Drain (hot chicken) | 2020 | (J) RTE and deli food factory | II | 204 | 1/2a | *fosX* | *actA, agrA, ami, aut, eut_operon, flaA, flgC, flgE, gadA, gadB, gadC, lap, lapB, oatA, oppA, orfX, orfZ, rli55, rli60, rsbv, biLE, bsh, btlB, chiA, clpB, clpc, clpe, clpp, codY, ctaP, ctsR, dal, degU, dltA, fbpA, fri, fur, gtcA, htq, hly, htrA, hupC, iap, inlA, inlB, inlC, inlF, inlH, inlJ, inlk, lgt, lhrC, lipA, lisK, lisR, lmo0514, lmo0610, lmo2026, lmo2085, lntA, lpeA, lplA1, lsp, mogR, mpl, mprf, murA, perR, pgdA, pgl, plcA, plcB, prfA, prsA2, pycA, recA, relA, secA2, sigB, sipX, sipZ, sod, srtA, srtB, stp, svpA, tcsA, tig, uHpt, virR*  (90 genes) | Inc18  Rep26 |
|  | Floor (dispatch area) | 2020 | (G) Uncooked and RTE meat factory | II | 204 | 1/2a | *fosX* | *actA, agrA, ami, aut, eut_operon, flaA, flgC, flgE, gadA, gadB, gadC, lap, lapB, oatA, oppA, orfX, orfZ, rli55, rli60, rsbv, biLE, bsh, btlB, chiA, clpB, clpc, clpe, clpp, codY, ctaP, ctsR, dal, degU, dltA, fbpA, fri, fur, gtcA, hfq, hly, htrA, hupC, iap, inlA, inlB, inlC, inlF, inlH, inlJ, inlk, lgt, lhrC, lipA, lisK, lisR, lmo0514, lmo0610, lmo2026, lmo2085, lntA, lpeA, lplA1, lsp, mogR, mpl, mprf, murA, perR, pgdA, pgl, plcA, plcB, prfA, prsA2, pycA, recA, relA, secA2, sigB, sipX, sipZ, sod, srtA, srtB, stp, svpA, tcsA, tig, uHpt, virR*  (90 genes) | Inc18  Rep26 |
| **Fresh produce** | Leeks | 2021 | (J) RTE and deli food factory | I | 1 | 4b | *fosX* | *agrA, eut_operon, flaA, flgC, gadB, gadC, gmar, lap, oatA, oppA, orfX, orfZ, rli55, rli60, rsbv, biLE, bsh, btlB, chiA, clpc, clpp, codY, ctaP, ctsR, degU, dltA, fri, fur, hfq, hly, htrA, hupC, iap, inlA, inlC, inlJ, lgt, lhrC, lipA, lisK, lisR, lntA, lpeA, lsp, mogR, mpl, mprf, murA, perR, pgdA, pgl, plcA, plcB, prfA, prsA2, pycA, relA, secA2, sigB, sipX, sipZ, sod, srtA, srtB, stp, svpA, tcsA, tig, uHpt, virR*  (70 genes) | None |
|  | Potato | 2018 | (K) RTE airline food factory | I | 2 | 4b | *fosX* | *agrA, eut_operon, flaA, flgC, gadB, gadC, gmar, lap, oatA, oppA, orfX, orfZ, rli55, rli60, rsbv, biLE, bsh, btlB, chiA, clpc, clpe, clpp, codY, ctaP, ctsR, degU, dltA, fri, fur, hfq, hly, htrA, hupC, iap, inlA, inlC, inlJ, lgt, lhrC, lipA, lisK, lisR, lntA, lpeA, lsp, mogR, mpl, mprf, murA, perR, pgdA, pgl, plcA, plcB, prfA, prsA2, pycA, relA, secA2, sigB, sipX, sipZ, sod, srtA, srtB, stp, svpA, tcsA, tig, uHpt, virR*  (71 genes) | None |
|  | Spinach | 2018 | (K) RTE airline food factory | I | 5 | 1/2b | *fosX* | *agrA, ami, aut, eut_operon, flaA, gadA, gadB, gadC, gmar, lap, oatA, oppA, orfX, orfZ, rli55, rli60, rsbv, biLE, bsh, btlB, chiA, clpc, clpp, codY, ctaP, ctsR, degU, dltA, fri, fur, gtcA, hfq, hly, htrA, hupC, iap, inlA, inlB, inlC, inlJ, lgt, lhrC, lipA, lisK, lisR, lpeA, lsp, mogR, mpl, mprf, murA, perR, pgdA, pgl, plcA, plcB, prfA, prsA2, pycA, relA, secA2, sigB, sipX, sipZ, sod, srtA, srtB, stp, svpA, tcsA, tig, uHpt, virR*  (73 genes) | Inc18  Rep25 |
|  | Coriander/cilantro | 2019 | (J) RTE and deli food factory | I | 5 | 1/2b | *fosX* | *agrA, ami, aut, eut_operon, flaA, gadA, gadB, gadC, gmar, lap, oatA, oppA, orfX, orfZ, rli55, rli60, rsbv, biLE, bsh, btlB, chiA, clpc, clpp, codY, ctaP, ctsR, degU, dltA, fri, fur, gtcA, hfq, hly, htrA, hupC, iap, inlA, inlB, inlC, inlJ, lgt, lhrC, lipA, lisK, lisR, lpeA, lsp, mogR, mpl, mprf, murA, perR, pgdA, pgl, plcA, plcB, prfA, prsA2, pycA, relA, secA2, sigB, sipX, sipZ, sod, srtA, srtB, stp, svpA, tcsA, tig, uHpt, virR*  (73 genes) | Inc18  Rep26 |
|  | Vegetable (pre-wash) | 2019 | (K) RTE airline food factory | I | 5 | 1/2b | *fosX* | *actA, agrA, ami, aut, eut_operon, flaA, flgC, flgE, gadA, gadB, gadC, gmar, lap, lapB, oatA, oppA, orfX, orfZ, rli55, rli60, rsbv, biLE, bsh, btlB, chiA, clpB, clpc, clpe, clpp, codY, ctaP, ctsR, dal, degU, dltA, fbpA, fri, fur, gtcA, hfq, hly, htrA, hupC, iap, inlA, inlB, inlC, inlF, inlH, inlJ, inlk, lgt, lhrC, lipA, lisK, lisR, lmo0514, lmo0610, lmo2026, lmo2085, lntA, lpeA, lsp, mogR, mpl, mprf, murA, perR, pgdA, pgl, plcA, plcB, prfA, prsA2, pycA, recA, relA, secA2, sigB, sipX, sipZ, sod, srtA, srtB, stp, svpA, tcsA, tig, uHpt, vip, virR*  (91 genes) | Inc18  Rep25 |
|  | Cucumber | 2019 | (J) RTE and deli food factory | II | 204 | 1/2a | *fosX* | *actA, agrA, ami, aut, eut_operon, flaA, flgC, flgE, gadA, gadB, gadC, lap, lapB, oatA, oppA, orfX, orfZ, rli55, rli60, rsbv, biLE, bsh, btlB, chiA, clpB, clpc, clpe, clpp, codY, ctaP, ctsR, dal, degU, dltA, fbpA, fri, fur, gtcA, hfq, hly, htrA, hupC, iap, inlA, inlB, inlC, inlF, inlH, inlJ, inlk, lgt, lhrC, lipA, lisK, lisR, lmo0514, lmo0610, lmo2026, lmo2085, lntA, lpeA, lplA1, lsp, mogR, mpl, mprf, murA, perR, pgdA, pgl, plcA, plcB, prfA, prsA2, pycA, recA, relA, secA2, sigB, sipX, sipZ, sod, srtA, srtB, stp, svpA, tcsA, tig, uHpt, virR*  (90 genes) | Inc18  Rep26 |
| **RTE hummus** | Hummus | 2019 | (J) RTE and deli food factory | I | 5 | 1/2b | *fosX*  *tetM* | *agrA, ami, aut, eut_operon, flaA, gadA, gadB, gadC, gmar, lap, oatA, oppA, orfX, orfZ, rli55, rli60, rsbv, biLE, bsh, btlB, chiA, clpc, clpp, codY, ctaP, ctsR, degU, dltA, fri, fur, gtcA, hfq, hly, htrA, hupC, iap, inlA, inlB, inlC, inlJ, lgt, lhrC, lipA, lisK, lisR, lpeA, lsp, mogR, mpl, mprf, murA, perR, pgdA, pgl, plcA, plcB, prfA, prsA2, pycA, relA, secA2, sigB, sipX, sipZ, sod, srtA, srtB, stp, svpA, tcsA, tig, uHpt, virR*  (73 genes) | Inc18  Rep26 |
|  | Hummus (coriander chilli) | 2019 | (J) RTE and deli food factory | I | 5 | 1/2b | *fosX*  *tetM* | *agrA, ami, aut, eut_operon, flaA, flgC, gadA, gadB, gadC, gmar, lap, oatA, oppA, orfX, orfZ, rli55, rli60, rsbv, biLE, bsh, btlB, chiA, clpc, clpp, codY, ctaP, ctsR, degU, dltA, fri, fur, gtcA, hfq, hly, htrA, hupC, iap, inlA, inlB, inlC, inlJ, lgt, lhrC, lipA, lisK, lisR, lpeA, lsp, mogR, mpl, mprf, murA, perR, pgdA, pgl, plcA, plcB, prfA, prsA2, pycA, relA, secA2, sigB, sipX, sipZ, sod, srtA, srtB, stp, svpA, tcsA, tig, uHpt, virR*  (74 genes) | Inc18  Rep26 |
|  | Smoked hummus | 2021 | (J) RTE and deli food factory | II | 101 | 1/2a | *fosX* | *actA, agrA, ami, aut, eut_operon, flaA, flgC, flgE, gadB, gadC, lap, lapB, oatA, oppA, orfX, orfZ, rli55, rli60, rsbv, biLE, bsh, btlB, chiA, clpc, clpe, clpp, codY, ctaP, ctsR, dal, degU, dltA, fri, fur, gtcA, hfq, hly, htrA, hupC, iap, inlA, inlB, inlC, inlF, inlH, inlJ, inlk, lgt, lhrC, lipA, lisK, lisR, lmo0514, lmo0610, lmo2085, lntA, lpeA, lplA1, lsp, mogR, mpl, mprf, murA, perR, pgdA, pgl, plcA, plcB, prfA, prsA2, pycA, recA, relA, secA2, sigB, sipX, sipZ, sod, srtA, srtB, stp, svpA, tcsA, tig, uHpt, vip, virR*  (87 genes) | Inc18  Rep25 |
|  | Smoked hummus | 2021 | (J) RTE and deli food factory | II | 101 | 1/2a | *fosX* | *actA, agrA, ami, aut, eut_operon, flaA, flgC, flgE, gadB, gadC, lap, lapB, oatA, oppA, orfX, orfZ, rli55, rli60, rsbv, biLE, bsh, btlB, chiA, clpc, clpe, clpp, codY, ctaP, ctsR, dal, degU, dltA, fri, fur, gtcA, hfq, hly, htrA, hupC, iap, inlA, inlB, inlC, inlF, inlH, inlJ, inlk, lgt, lhrC, lipA, lisK, lisR, lmo0514, lmo0610, lmo2085, lntA, lpeA, lplA1, lsp, mogR, mpl, mprf, murA, perR, pgdA, pgl, plcA, plcB, prfA, prsA2, pycA, recA, relA, secA2, sigB, sipX, sipZ, sod, srtA, srtB, stp, svpA, tcsA, tig, uHpt, vip, virR*  (87 genes) | Inc18  Rep25 |
|  | Hummus | 2018 | (J) RTE and deli food factory | II | 121 | 1/2a | *fosX* | *actA, agrA, ami, aut, eut_operon, flaA, flgC, flgE, gadB, gadC, lap, lapB, oatA, oppA, orfX, orfZ, rli55, rli60, rsbv, biLE, bsh, btlB, chiA, clpB, clpc, clpe, clpp, codY, ctaP, ctsR, dal, degU, dltA, fbpA, fri, fur, gtcA, hfq, hly, htrA, hupC, iap, inlA, inlB, inlC, inlH, inlJ, inlk, lgt, lhrC, lipA, lisK, lisR, lmo0514, lmo0610, lmo2085, lntA, lpeA, lplA1, lsp, mogR, mpl, mprf, murA, perR, pgdA, pgl, plcA, plcB, prfA, prsA2, pycA, recA, relA, secA2, sigB, sipX, sipZ, sod, srtA, srtB, stp, svpA, tcsA, tig, uHpt, vip, virR*  (88 genes) | Inc18  Rep26 |
|  | Hummus (orange) | 2019 | (J) RTE and deli food factory | II | 121 | 1/2a | *fosX* | *actA, agrA, ami, aut, eut_operon, flaA, flgC, flgE, gadB, gadC, lap, lapB, oatA, oppA, orfX, orfZ, rli55, rli60, rsbv, biLE, bsh, btlB, chiA, clpB, clpc, clpe, clpp, codY, ctaP, ctsR, degU, dltA, fbpA, fri, fur, gtcA, hfq, hly, htrA, hupC, iap, inlA, inlB, inlC, inlJ, inlk, lgt, lhrC, lipA, lisK, lisR, lmo0514, lmo0610, lmo2085, lntA, lpeA, lplA1, lsp, mogR, mpl, mprf, murA, perR, pgdA, pgl, plcA, plcB, prfA, prsA2, pycA, recA, relA, secA2, sigB, sipX, sipZ, sod, srtA, srtB, stp, svpA, tcsA, tig, uHpt, vip, virR*  (86 genes) | Inc18  Rep26 |

Factory origin information was requested from Microchem Lab Services (Pty) Ltd (maintaining anonymity) after WGS and bioinformatic analysis for discussion purposes.

**^*^**Genes detected using the Centre for Genomic Epidemiology (CGE) VirulenceFinder 2.0.
