# Supplementary material for: Profiling Listeria monocytogenes in Hummus, Fresh Produce, and Food Processing Environments in the Western Cape, South Africa
Source: Microbiologyopen. 2025 Sep 8;14(5):e70060. doi: 10.1002/mbo3.70060 (PMC12417568; doi:10.1002/mbo3.70060)
Supplement: Supplementary file 4 — Supporting Table 3: The basic functions of virulence genes detected in L. monocytogenes isolates in this study (Dussurget 2008). [file MBO3-14-e70060-s001.docx]

**Supplementary Table 3:** The basic functions of virulence genes detected in *L. monocytogenes* isolates in this study (Dussurget, 2008)

| **Basic function** | **Genes** |
| --- | --- |
| Regulation of virulence gene expression | *prfA, sigB, mogR, ctsR, per, fur, lisR, lisK, agrA, degU, stp, hfq* |
| Adaption to host extracellular compartments | *gad, bsh, biLE, btlB, oppA* |
| Vacuole escape | *plcA, plcB, mpl, hly* |
| Intracellular survival and multiplication | *lsp, sipX, sipZ, fri, hupC, relA, lgt* |
| Adhesion | *ami, dltA, fbpA, flaA* |
| Invasion | *Iap, inlA, inlB, srtA, srtB, aut, vip, lpeA* |
| Cell to cell spread | *actA, secA2* |
